# Pattern of respiratory diseases in children presenting to the paediatric emergency unit of the University of Nigeria Teaching Hospital, Enugu: a case series report

**DOI:** 10.1186/1471-2466-14-101

**Published:** 2014-06-10

**Authors:** Tagbo Oguonu, Chikaodinaka Adaeze Ayuk, Benedict Onyeka Edelu, Ikenna Kingsley Ndu

**Affiliations:** 1 Department of Paediatrics, University of Nigeria Teaching Hospital, Enugu, Nigeria; 2 Department of Paediatrics, Enugu State University of Technology Teaching Hospital Enugu, Enugu, Nigeria

**Keywords:** Children, Enugu, Pattern, Respiratory diseases

## Abstract

**Background:**

Respiratory diseases are one of the causes of childhood morbidity and mortality as well as hospitalization globally. The patterns of different respiratory illnesses in several parts of the world have been reported but there are few on the combined burden of the diseases. Determination of the burden of respiratory diseases as a group will help ascertain their collective impact on the health systems in order to develop intervention measures.

**Methods:**

Data from case notes of children with respiratory diseases admitted to the University of Nigeria Teaching Hospital Enugu, Nigeria over a six year period were extracted. Age, gender, admission rates, types of respiratory illness, duration of admission, season of presentation and outcome were analysed. Descriptive and inferential (Chi square) statistics were used to describe the various disease types and ascertain association of the disease outcome, seasonal pattern with the types of diseases.

**Results:**

Of the total of 8974 children admissions, 2214 (24.7%) were due to respiratory diseases. The mean age of all the children with respiratory diseases was 3.3 years (SD 3.9). Communicable diseases were the common cause of admission cases throughout the seasons, p < 0.001. The highest admission rates were for pneumonia, (34.0%), acute bronchial asthma, (27.7%) and rhinosinusitis (14.6%) p < 0.001. The frequency of respiratory disease decreases with age and children less than five years of age and of low socio-economic status were commonly affected, p = 0.01. The median duration of hospital stay was two days [range 1 to 8 days], children less than five years old and those of low socio-economic status, spent more than four days (p = 0.01 and p < 0.001 respectively). The all-cause mortality was 0.5% (11/2214) of which 81.8% (9/11) was due to pneumonia.

**Conclusions:**

Respiratory diseases constitute a significant burden of childhood illnesses in our centre. Efforts are required to reduce the impact as part of the steps towards the achievement of the Millennium Development Goals.

## Background

Respiratory diseases remain a major cause of morbidity and mortality in children [1-3] especially among children less than five years old [1].The spectrum of respiratory illnesses is wide and includes diseases of upper and lower airways, communicable and non-communicable types. The variations in pattern of morbidity and mortality of respiratory illnesses may be affected by different home/environmental and climatic variations in different parts of the world [4,5]. The World Health Organization (WHO) estimates that approximately 10.6 million children under five years of age die each year, acute respiratory infection (ARI), especially pneumonia [1], contributes about 19% of the total number of deaths.

Epidemiological studies have shown different estimates of the burden of respiratory diseases in different countries. In the US respiratory diseases in children are responsible for 25% of hospital admissions [6] while in the United Kingdom [7] and continental Europe [8] respiratory diseases contribute to 25% and 13% of hospital admissions among the paediatric age groups respectively. Within the African sub-region the burden of the diseases is not well defined due to paucity of data [2]. In Malawi, 298/1000 cases of admission were due to respiratory diseases in children [9].

More often the studies on respiratory diseases are on the specific illnesses that cause morbidity and mortality such that the combined pattern of the respiratory diseases is ignored. The value of data on respiratory diseases is that it enhances knowledge on the types and burden of the categories of diseases that affect the respiratory system. This will help in developing intervention measures both at the institutional and national levels. Data is scarce; there are few published works on the combined burden of respiratory diseases in children in Nigeria and other developing countries [2,9].

This study was done to ascertain the pattern and outcome of respiratory diseases in children presenting to the paediatric emergency unit of the University of Nigeria Teaching Hospital, Enugu. This will form an epidemiological database for further studies of risk factors of respiratory diseases and also help in allocation of scare resources towards those respiratory diseases with significant burden.

## Methods

Information from case notes of children that presented at the Children Emergency Unit of the University of Nigeria Teaching Hospital (UNTH), Ituku/Ozalla, Enugu, Nigeria from January 2007 to December 2012 was reviewed. The Children Emergency Unit is a 24-bed facility with basic equipment for resuscitation and treatment, manned by a full complement of staff that include interns, paediatric residents, consultant paediatricians, nurses of various cadres, laboratory assistants and records clerks.

For the review of the cases the ward register was used to compile the list of patients attended to in the unit. Subsequently the notes of patients with respiratory diseases were then extracted and examined for relevant information. Inclusion criteria were based on the clinicians’ diagnoses of the respective respiratory diseases using the clinical features and available laboratory results. Other relevant information retrieved included age, sex, parent’s occupation and educational qualifications, place of domicile, month/year of presentation, duration of hospitalization and admission outcome. Ethical approval was obtained from the Health Research and Ethics Committee of the University of Nigeria Teaching Hospital (UNTH), Ituku/Ozalla, Enugu.

Data was entered into Microsoft Excel® (2010, Microsoft Corporation, Redmond Washington USA) and analyses subsequently done with Statistical Package for Social Sciences (SPSS) version 20 (2011, IBM Inc. Chicago Illinois, USA). The variables were: age, gender, month/season of presentation and socioeconomic status, type of respiratory illness, length of hospital stay and admission outcome. Considering that respiratory illnesses mainly affect under-fives’ [1] age was further categorized to below 5 years, 5 to 9.9 years and ≥ 10 years. To assess duration of stay for respiratory illnesses, length of hospital stay was categorized into stay of less than 5 days and 5 or more days. The socio economic status (SEC) was determined with caregiver/parents’ education attainment and occupation as described by Oyedeji [10] and were grouped into high, middle and low socioeconomic classes. Period of presentation was grouped to correspond to the prevalent seasons in Nigeria namely: the rainy season (April to September) and dry season (October to March).

Frequencies of the variables were performed for initial descriptive statistics and data cleaning. Analyses of the variables were performed to determine their distribution types and the statistical tools that were applicable. Of the subjects’ characteristics; age was the only variable that had a normal distribution while the others which were categorized were of non-Gaussian distribution. To test the association between variables, the chi-square test was used for nominal variables and the Spearman’s correlation for ordinal variables. To evaluate the possible determinants of admissions (eg repeated admissions) multivariate analysis was performed. Statistical significant value was set at p value of ≤ 0.05 with confidence interval of 95%.

## Results

There was a total of 8974 children admissions to the emergency unit during the period of review: January 2007 and December 2012. Of these admissions, 2214 (24.7%) were due to respiratory illnesses (Table 1).

**Table 1:** Annual distribution of admissions in the emergency unit

**Year**	**Number of all admissions into the emergency unit**	**Number of cases with respiratory illnesses n (%)**
2007	993	377 (37.9)
2008	1383	364 (26.3)
2009	1138	274 (24.1)
2010	2090	438 (21.0)
2011	1519	407 (26.8)
2012	1851	354 (19.1)
**Total**	8974	2214 (24.7)

### General characteristics

The mean age of all the children with respiratory diseases was 3.3 years (SD 3.9), with a maximum age 20 years and minimum of one day. Sixty one percent of study participants were male. Their age distribution showed that 75.2% were less than five years old, 13.7% were between 5 and 9.9 years old, while 9.8% were 10 years and above. There were 1584 (71.6%) subjects who had their SEC listed, out of which 11.1% children were from high-income families while 44.9% and 44.1% were from middle, low-income families respectively and 29.4% unspecified.

### Illness type

There were 22 types/categories of respiratory disease among those admitted comprising of 67.6% and 32.4% due to communicable and non-communicable causes respectively, (Table 2). The three common respiratory illnesses admitted during the period were pneumonia (34.0%), acute bronchial asthma (27.7%) and rhinosinusitis (14.6%), these were more prevalent among the under-five age group (p < 0.001) (Table 3). Of the subjects that had socioeconomic class specified pneumonia, coryza, and acute chest syndrome were more among the low income group, while asthma and hydrocarbon poisoning were more prevalent among children of the middle class p < 0.001.

**Table 2:** Frequency of Respiratory illnesses by categories/ types

**Category**	**Diagnosis**		**Frequency (n) (%)**
Communicable	Pneumonia	753	50.4
	Rhinosinusitis	324	21.7
	Bronchiolitis	223	14.9
	Adenotonsilitis	92	6.2
	Acute otitis media	54	3.6
	Pulmonary tuberculosis	27	1.8
	Croup	11	0.7
	Suspected pertussis	6	0.4
	Parotitis	3	0.2
	Acute epiglottitis	1	0.1
	Bronchiectasis	1	0.1
	**Total**	1495	100.0
Non-Communicable	Acute bronchial asthma	608	84.6
	Foreign body inhalation (non-hydrocarbon)	59	8.2
	Kerosene aspiration	25	3.5
	Acute chest syndrome	13	1.8
	Laryngomalacia	7	1.0
	Epistaxis	2	0.3
	Nasal polyp	2	0.3
	Traumatic ottorhea	1	0.1
	Choanal atresia	1	0.1
	Meconium aspiration	1	0.1
	**Total**	719	100.0

**Table 3:** Frequency of respiratory illnesses by age groups

**Respiratory disease**	**Age group (years)**			**Missing age**	**Total n (100%)**
	**0 to <5 n (%)**	**≥ 5 to < 10 n (%)**	**≥10 n (%)**		
Acute bronchial asthma	289 (47.5)	175 (27.8)	135 (22.2)	9 (1.5)	608
Acute chest syndrome	4 (30.8)	3 (23.1)	6 (46.1)	0 (0.0)	13
Acute epiglottitis	1(100.0)	0(0.0)	0 (0.0)	0 (0.0)	1
Acute otitis media	45(83.3)	5(9.3)	3 (5.6)	1 (1.8)	54
Adenotonsilitis	60 (65.2)	22(24.0)	5 (5.4)	5 (5.4)	92
Bronchiectasis	0 (0.0)	0(0.0)	1(100.0)	0 (0.0)	1
Bronchiolitis	220 (98.7)	3(1.3)	0 (0.0)	0 (0.0)	223
Choanal atresia	1 (100.0)	0(0.0)	0 (0.0)	0 (0.0)	1
Croup	10 (90.9)	1 (9.1)	0 (0.0)	0 (0.0)	11
Epistaxis	1 (50.0)	1 (50.0)	0 (0.0)	0 (0.0)	2
Foreign body inhalation (non-hydrocarbon)	45(76.3)	8 (13.5)	1 (1.7)	5 (8.5)	59
Laryngomalacia	7(100.0)	0 (0.0)	0 (0.0)	0 (0.0)	7
Meconium aspiration	1(100.0)	0(0.0)	0 (0.0)	0 (0.0)	1
Nasal polyp	1 (50.0)	1(50.0)	0 (0.0)	0 (0.0)	2
Parotitis	3 (100.0)	0 (0.0)	0 (0.0)	0 (0.0)	3
Pertussis	5 (83.3)	0 (0.0)	0 (0.0)	1(16.7)	6
Pneumonia	656 (87.1)	52 (6.9)	41(5.4)	4 (0.6)	753
Pulmonary tuberculosis	13 (48.2)	5 (18.5)	9 (33.3)	0 (0.0)	27
Hydrocarbon aspiration	23 (92.0)	2 (8.0)	0 (0.0)	0 (0.0)	25
Rhinosinusitis	279 (86.1)	26 (8.0)	12 (3.7)	7 (2.2)	323
Traumatic ottorhea	1 (100.0)	0 (0.0)	0 (0.0)	0 (0.0)	1
Total	1665 (75.2)	304 (13.7)	213 (9.6)	32 (1.5)	2214

### Seasonal presentation

**Figure 1:**
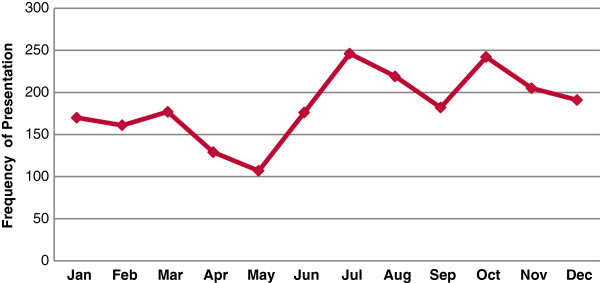
Monthly distribution pattern of admitted cases.

Collectively there was a near equal presentation of cases in the rainy and dry seasons: 51.9% and 48.1% respectively, the highest admissions were in July (11.2%) and October (10.9%) (Figure 1). Of the two categories of diseases (communicable and non-communicable), admissions for communicable diseases were the main cause of admissions throughout the seasons especially during the dry season. 63.57% and 71.37% respectively p < 0.001, (Table 4). However, there were seasonal variations in pattern of admissions for specific illnesses; children with asthma were seen more during the rainy (55.1%) than dry season (44.9%), while pneumonia, acute otitis media, and bronchiolitis were admitted more during the dry season: 52.59%, 75.2%, and 52.02% respectively.

**Table 4:** Seasonal distribution of the categories of respiratory diseases admitted

**Category of respiratory disease**	**Season of the year**		**Total n**
	**Rainy n (%)**	**Dry n (%)**	
Communicable	677 (63.57)	820 (71.37)	1497
Non-communicable	388 (36.43)	329 (28.63)	717
Total (%)	1065 (100)	1149 (100)	2214 (100)

χ^2^15.35; p <0.001.

### Duration of admission

The median duration of admission was two days [range 1 to 8 days, mid- quartile: 1 to 3 days], 12.7% of the children were admitted for more than four days. Children less than five years were about three times more likely to stay more than four days than the other age groups (p < 0.001, OR = 2.69, C.I = 1.84 to 3.94). The proportions of children from the different socioeconomic classes who stayed longer than four days were 18.4%, 16.2%, 14.5%, for the low, high and middle income groups respectively, p < 0.001. Repeated admissions were noted among 21.2% of the children; 14.4% were those less than 5 years and 7.8% were older than 5 years, p < 0.01. For possible predictors of repeated visits, the tests of the model coefficients was statistically significant (chi square 145.5, p < 0.001 with df =6. The Nagelkerke’s R^2^ of 0.099 showed a weak relationship between the prediction and the grouping. Only disease types (Non-communicable) and high socio-economic status were significant predictors of repeated admissions, (OR= 0.37, CI=0.0.30 to 0.47, p < 0.001 and OR = 0.43, CI= 0.32 to 0.58, p < 0.001 respectively). The age group and season of presentation made no significant contribution to the prediction.

### Admission outcome

Majority, 91.9% of cases admitted were discharged home, 7.6% were transferred to inpatient wards while death was recorded in 0.5% (11/2214) of the children. Most of the deaths 72.7% (8/11) occurred in children less than 5 years old. The patients from the low socio-economic class had the least proportion of discharged cases and contributed disproportionately to the number of deaths, eight out of eleven cases, p < 0.001. Nine of the eleven recorded deaths were those diagnosed with pneumonia, p < 0.001, Table 5.

**Table 5:** Admission outcome of respiratory illnesses

**Respiratory illness**	**Discharged/inpatient transfer n (%)**	**Died n (%)**	**Total**	**Case fatality rates (%)**
Acute bronchial asthma	607 (99.8)	1	608	0.2
Epistaxis	1 (50)	1	2	50
Pneumonia	744 (98.8)	9	753	1.2
Others	855 (100)	0	851	0
Total	2203	11	2214	0.5

## Discussion

This study has shown that respiratory diseases are common indications for admissions of children in Enugu particularly among those under five years of age. The acute illnesses and communicable diseases predominate. The yearly prevalence of the respiratory diseases over the study period has been consistently high but with no particular pattern over the years irrespective of the number of hospitalized patients, however, the last year of the study, 2011, showed a decline. There is no clear reason for the observation but may be due to the noted decline in incidence of pneumonia and other infectious diseases [11].

According to various epidemiological (hospital-based) studies the communicable diseases of the respiratory system cause significant morbidity and mortality particularly in children less than five years old. Hospital-based case series studies in Nigeria; PortHarcourt [12], Benin [13], although not combined studies of respiratory diseases but collation of the findings, showed similar pattern of diseases and proportions. In a study of admissions in hospitals in Hong Kong [14], respiratory disorders constituted 37.5% of all diagnoses, with upper respiratory infections and pneumonia comprising 30.1% and 20.9% respectively.

Pneumonia, bronchiolitis, rhino sinusitis made up 96.8% of all communicable respiratory illnesses and the most common of all the respiratory diseases, similar to findings of other workers [2-4,12]. The role of pneumonia as a major contributor to childhood respiratory disease burden has continued to be a public health concern, particularly in the developing parts of the world. [11,15] In contrast, the reports in Norway by Kervold et al. [16] showed a preponderance of communicable diseases affecting the upper airways.

Asthma, foreign body inhalation/poisoning and acute chest syndrome of sickle cell disease constitute the topmost cases of non-communicable diseases admitted in our study. Asthma cases were a major cause of hospitalization of children [17,18] responsible for a third of overall cases admitted in our study. A comparison of the currently observed incidence of asthma with a previous one from our centre ten years ago showed an increase of 3.5% [19]. The observation underscores the recently reported increase in the prevalence of asthma in developing countries [20,21]. The reasons for this upsurge are not very clear and have been attributed to life style changes [22], increased awareness and ability to diagnose by physicians [20]. Related to this is probably the failure to achieve control of asthma among those with persistent disease consequently experiencing frequent exacerbation and admissions. The need to improve the case management of asthma and provision of affordable medications for the children with the disease cannot be over emphasized if there should be any appreciable impact on asthma burden in our setting.

Acute chest syndrome (ACS) is an acute episode of respiratory distress among sickle cell anaemia (SCA) patients which leads to hospital admissions and can be fatal [23]. It is a primary cause of admission in children with sickle cell anaemia [24] as was noted in our study. Access and facility as a tertiary centre may be responsible for the admission rate observed in our series. Related to this is the high burden of the sickle cell disease in Nigeria [23] which will translate to higher number of children with associated respiratory complications. The unstable status noted among children with sickle cell anaemia could possibly be a contributory factor to admission rates observed. Studies [23,25] in the United States show that acute chest syndrome causes up to 25.3 episodes/100 patient years with high morbidity among children. The findings in our study could then spur some interest in improved case identification and early effective treatment among this group of patients.

Our patients’ socio-demographic characteristics are similar to findings by other studies in developing countries; the preponderance of pneumonia among the low socio-economic class is the observation in most countries in the developing world in contrast to what is observed in the developed world. This is related to the factors associated with low socio-economic class, which are poverty, overcrowded habitations; factors that enhance the spread of infections [25-27].The combination of the factors and the high incidence noted in this study portray the inadequacy of ameliorating measures and demands more effort in control measures such as provision of immunization and health education to reduce the burden.

Cases due to accidents and poisoning particularly foreign substance (kerosene) inhalation was noted in a substantial number of admissions among children less than five year olds. Kerosene is a common fuel for cooking among the middle and low socio-economic classes. Storage problems due to overcrowding in the households make the substance readily available to cause harm to children. The proportion of cases with kerosene poisoning although small illustrates the lingering problem of the inadequacy of health education in domestic accident prevention and the failure of the institutions (family and government) to provide protection for the less privileged by legislation or improved housing.

Tupasi et al. [28] identified low socioeconomic group and age less than one year as risk factors for ARI in the Philippines. Berman [29] had noted that among other factors crowding in households and young age contribute to the incidence and severity of lower respiratory infections in developing countries. The role of socioeconomic status in disease prevalence has been noted by so many studies [10,27,28] with more impact noted among the low class. The factor usually attributed to this is the multiplier impact of poverty on health seeking behavior, ignorance and lack of financial resources.

The significance of age as a contributory factor to admissions due to respiratory diseases has also been observed in this and other studies. Chang in Australia [30] had a median age of 1.8 years similar to ours also Uijen et al. [8] in Netherlands and Ugwu and co-workers [31] in Niger-Delta region of Nigeria both noted preponderance of males and children younger than five years. This highlights the vulnerability of the under-five age group to respiratory illness that may be related to less mature immune systems as well as less compliant lungs which increase their susceptibility to infections and other airway diseases [32] resulting in relatively increased morbidity and hospital presentation and admissions.

The observed seasonal variation in the incidence of the various respiratory diseases may be attributed to the environmental factors related to the diseases. Majority of cases of asthma admitted in the rainy season are probably due to the effect of cold weather as an etiological factor of the disease. In the northern hemisphere admission for cases of asthma are known to increase during the cold seasons. [33-35] The dissemination of pollen, the close indoor activities necessitated by the cold weather have been attributed to this prevalence.

Dry seasons are associated with dusty environment which tend to promote sporulation and transmission of pathogen particles with resultant inhalation and infection of susceptible individuals. Thus the increased incidence of respiratory infections during the dry season is agreeable with this observation. Desalu [36] reported a seasonal variation of respiratory diseases in Nigeria while Fagbule and coworkers [37] in contrast observed no seasonal variation in the incidence of respiratory diseases. Gbadero et al. [38] observed a preponderance of asthma admissions in the dry season in contrast to our finding. All the reports are case series studies which are prone to variations in definition/diagnosis as well as data retrieval which may be responsible for the differences observed. Nevertheless this may strengthen the case for more robust research in our environment to identify seasonal disease-modifying factors associated with hospitalization of respiratory diseases. Seasonal variation as observed will enable our facility and probably others in the region to plan for both preventive and other intervention measures targeted at season specific incidence of the various diseases in order to reduce the morbidity and mortality rates.

The proportion of deaths of less than ten per thousand for respiratory diseases is small in comparison with published mortality rates [39,40]. There is also dissimilarity with findings in other developing countries. A critical review will show that although the overall rate is low the preponderance of pneumonia in all-cause mortality is also high and similar to reports from other centers irrespective of facility [41]. Low all cause-mortality figures obtained in this study are expected considering the nature of the facility. In similar facilities there have been different mortality figures reported. The asthma case fatality rate of 0.2% is in consonance with reported rates [42] of asthma in so many other populations. Death by asthma is rare and expectedly so in a hospital based study, which may differ from what could be obtained in a population study.

### Limitations of study

Although a hospital based study provides a means of audit of the morbidity and mortality in the hospital setting as well as mirrors the probable state of diseases in the general population it still lacks the representation of the exact existence of the subject being studied. This represents one of the limitations of the study. The study design (case series) equally creates the limitation on data collection. Nevertheless these do not remove the essence of the study objective but will serve as the basis for further robust study on the subject. Regarding the data, the lack of information on comorbidities may affect the assessment of the outcome of the various diseases.

## Conclusions

What our findings show is that despite the best efforts with the different intervention programs recommended by multilateral agencies such as the WHO UNICEF [14,15] respiratory diseases still constitute some significant burden to children. There are similarities in proportion and variation in the types of diseases in the low/middle and the high income countries. It thus requires more effort among all concerned to reduce the disease burden and should be directed at improvement in the intervention methods and or more commitment by the stakeholders in implementation of the existing methods.

Attention should be paid to respiratory diseases in children to reduce the morbidity in the population. A comprehensive study in the community on the epidemiological factors associated with morbidity and mortality should be undertaken in order to determine the prevalence and plan interventions on management of the diseases.

## Abbreviations

ACS: Acute chest syndrome; ARI: Acute respiratory infections; SCA: Sickle cell anemia; SEC: Socioeconomic class; SPSS: Statistical package for social sciences; UNICEF: United Nations Children Education Fund; UNTH: University of Nigeria Teaching Hospital; US: United States; WHO: World Health Organization.

## Competing interests

The authors declare that they have no competing interests.

## Authors’ contributions

TO conceived of the study participated in its design and coordination and helped to draft the manuscript and the statistical analysis. AAC designed the manuscript template and coordinated the manuscript draft. She also did the statistical analysis upon which others made contributions. BOE participated in the study design contributed in the draft of the manuscript and helped in the statistical analysis. KIN collected and entered the data, participated in the manuscript drafting and contributed in the statistical analysis. All authors read and approved the final manuscript.

## Authors’ information

TO FMCPaed, Consultant Paediatrician in-charge of the Children Emergency Unit, UNTH, Ituku-Ozalla, Enugu. ACA- FMCPaed, Consultant Paediatrician, UNTH, Ituku-Ozalla, Enugu. BOE FMCPaed, Consultant Paediatrician, UNTH, Ituku-Ozalla, Enugu. IKN – FWACP, Consultant Paediatrician in-charge of the Children Emergency Unit, Enugu State University Teaching Hospital, Enugu.

## Pre-publication history

The pre-publication history for this paper can be accessed here:

http://www.biomedcentral.com/1471-2466/14/101/prepub

## Reason why the article should be published

This article highlights the burden and pattern of respiratory diseases in our centre. It will help to develop institutional or national interventional measures.

The article will also help to form an epidemiological database for further studies as there is paucity of publications on the burden of respiratory diseases in children

Data is scarce with few published works on the combined burden of respiratory diseases in children in Nigeria and other developing countries, compared to data on individual respiratory diseases, hence the importance of this publication.
